# Cognitive Impairment Before Intracerebral Hemorrhage Is Associated With Cerebral Amyloid Angiopathy

**DOI:** 10.1161/STROKEAHA.117.019409

**Published:** 2017-12-15

**Authors:** Gargi Banerjee, Duncan Wilson, Gareth Ambler, Karen Osei-Bonsu Appiah, Clare Shakeshaft, Surabhika Lunawat, Hannah Cohen, Tarek Yousry, Gregory Y.H. Lip, Keith W. Muir, Martin M. Brown, Rustam Al-Shahi Salman, Hans Rolf Jäger, David J. Werring

**Affiliations:** From the UCL Stroke Research Centre (G.B., D.W., K.O.-B.A., C.S., S.L., M.M.B., D.J.W.) and Lysholm Department of Neuroradiology and the Neuroradiological Academic Unit (T.Y., H.R.J.), Department of Brain Repair and Rehabilitation, UCL Institute of Neurology, and the National Hospital for Neurology and Neurosurgery, London, United Kingdom; Department of Statistical Science (G.A.) and Haemostasis Research Unit, Department of Haematology (H.C.), University College London, United Kingdom; University of Birmingham Institute of Cardiovascular Sciences, City Hospital, United Kingdom (G.Y.H.L.); Institute of Neuroscience & Psychology, University of Glasgow, Queen Elizabeth University Hospital, United Kingdom (K.W.M.); and Centre for Clinical Brain Sciences, School of Clinical Sciences, University of Edinburgh, United Kingdom (R.A.-S.S.).

**Keywords:** cerebral amyloid angiopathy, cerebral hemorrhage, cerebral small vessel diseases, cognitive dysfunction, prevalence, siderosis

## Abstract

**Background and Purpose—:**

Although the association between cerebral amyloid angiopathy (CAA) and cognitive impairment is increasingly recognized, it is not clear whether this is because of the impact of recurrent intracerebral hemorrhage (ICH) events, disruptions caused by cerebral small vessel damage, or both. We investigated this by considering whether cognitive impairment before ICH was associated with neuroimaging features of CAA on magnetic resonance imaging.

**Methods—:**

We studied 166 patients with neuroimaging-confirmed ICH recruited to a prospective multicentre observational study. Preexisting cognitive impairment was determined using the Informant Questionnaire on Cognitive Decline in the Elderly (IQCODE). Magnetic resonance imaging markers of cerebral small vessel disease, including CAA, were rated by trained observers according to consensus guidelines.

**Results—:**

The prevalence of cognitive impairment before ICH was 24.7% (n=41) and, in adjusted analyses, was associated with fulfilling the modified Boston criteria for probable CAA at presentation (odds ratio, 4.01; 95% confidence interval, 1.53–10.51; *P*=0.005) and a higher composite CAA score (for each point increase, odds ratio, 1.42; 95% confidence interval, 1.03–1.97; *P*=0.033). We also found independent associations between pre-ICH cognitive decline and the presence of cortical superficial siderosis, strictly lobar microbleeds, and lobar ICH location, but not with other neuroimaging markers, or a composite small vessel disease score.

**Conclusions—:**

CAA (defined using magnetic resonance imaging markers) is associated with cognitive decline before symptomatic ICH. This provides evidence that small vessel disruption in CAA makes an independent contribution to cognitive impairment, in addition to effects due to brain injury caused directly by ICH.

**Clinical Trial Registration—:**

URL: https://www.clinicaltrials.gov. Unique identifier: NCT02513316.

## Abstract 2

Supplemental Digital Content is available in the text.

Although the associations between dementia and ischemic stroke have been comprehensively described,^1^ fewer data are available for spontaneous intracerebral hemorrhage (ICH), in part because of its high case fatality.^2,3^ Cognitive impairment often develops in survivors of ICH who were previously dementia free, particularly if the ICH is lobar, and has been associated with baseline neuroimaging markers of cerebral amyloid angiopathy (CAA).^2^ In those presenting with ICH, cognitive impairment before the event is common, with an estimated pooled incidence of 16.7%,^4^ suggesting that the underlying neurovascular and neuropathological processes that result in cognitive impairment after ICH might already be present at the time of initial presentation with ICH.^2,4,5^ However, it is not clear to what extent subsequent cognitive impairment after ICH is mediated by direct damage from the index ICH, the effects of recurrent ICH, or the impact of the underlying small vessel disease (SVD)^2,4^; understanding the contribution of these mechanisms is potentially important in developing rational dementia prevention strategies.

We therefore investigated whether neuroimaging evidence of CAA (specifically, meeting the modified Boston criteria for probable CAA^6^ at presentation, and increases in a composite CAA score^7^) was associated with the presence of cognitive impairment before ICH. We then performed further analyses investigating the associations between individual magnetic resonance imaging (MRI) neuroimaging markers of SVD and cognitive impairment before ICH.

## Materials and Methods

### Patient Selection

We included patients recruited to a prospective multicentre observational cohort study of symptomatic patients with confirmed ICH (The Clinical Relevance of Microbleeds In Stroke Study; CROMIS-2). Those aged ≥18 years with an ICH confirmed on brain imaging (either computed tomography or MRI) were eligible, providing that there was no evidence that the ICH was because of an underlying structural cause or secondary to head trauma. This study has been preregistered, and the full details of the study protocol have been published previously.^8^ The study was approved by the National Research Ethics Service (IRAS reference 10/H0716/61). Written informed consent was obtained from each patient. The primary and substudy analyses for the CROMIS-2 study are ongoing; once all of these analyses are completed, the CROMIS-2 Steering Committee will consider applications from other researchers for access to anonymized source data.

The Informant Questionnaire for Cognitive Decline in the Elderly (IQCODE) is a validated questionnaire given to a patient’s family member or caregiver which aims to establish whether there have been specific changes in cognitive and functional performance over the preceding 10-year time period.^9–11^ Specifically, the informant was asked to compare the patient’s performance from 10 years ago with their performance just before their stroke. The 16-item IQCODE was requested for all participants (score range, 1.0–5.0); this version of the IQCODE has been reported to have similar accuracy to the original 26-item version.^10^ We defined pre-ICH cognitive impairment as an IQCODE score of >3.3, based on previously reported pooled sensitivity and specificity values for detecting cognitive impairment from a meta-analysis investigating IQCODE accuracy in a general hospital setting.^10^

For inclusion in the final analysis, it was necessary for patients to have an IQCODE from the time of their admission, together with the MRI sequences needed for imaging analysis (described below).

### Imaging Acquisition and Analysis

Imaging was undertaken at each study center according to local protocols, and all brain imaging performed as part of the participant’s standard clinical care was sent to the study’s coordinating center in anonymized DICOM format.

Imaging analysis was performed by 2 clinical research associates (D.W., G.B.) and 2 MSc students (K.O.-B.A, S.L.), all of whom were trained in neuroimaging rating and blinded to the participant clinical details. All structural imaging markers of cerebral SVD were rated in accordance with the Standards for Reporting Vascular Changes on Neuroimaging consensus criteria.^12^ Only those with an available MRI and all of the necessary sequences for cerebral SVD rating (ie, axial T2, axial or coronal fluid-attenuated inversion recovery (FLAIR), and a blood-sensitive sequence) were included in the neuroimaging marker analysis.

Lacunes were identified and counted (D.W.) on T2 and FLAIR sequences.^12^ Cerebral microbleeds were rated (D.W.) using blood-sensitive (T2* weighted or susceptibility weighted images) sequences and the validated Microbleed Anatomical Rating Scale.^13^ MRI-visible perivascular spaces (PVS) in the centrum semiovale (CSO-PVS) and basal ganglia (BG-PVS) were defined and rated (G.B.) on T2 and FLAIR sequences using a validated 4-point visual rating scale^12,14,15^on a single predefined slice (first slice above the anterior commissure for the basal ganglia, and the first slice above the level of the lateral ventricles for the centrum semiovale). The hemisphere contralateral to the ICH was preferentially rated. White matter hyperintensities (WMH; also termed leukoaraiosis) were rated (K.O.-B.A.) on T2 and FLAIR sequences using the Fazekas scale.^16,17^ Cortical superficial siderosis (cSS) was identified on blood-sensitive sequences and classified (D.W.) as either focal (involving ≤3 sulci) or disseminated (involving ≥4 sulci), in keeping with previously described terminology.^18^ Medial temporal atrophy (MTA) was rated (G.B.) on T1 or FLAIR coronal images using the Scheltens visual scale.^19,20^ Global cortical atrophy (GCA) was rated (G.B.) using the Pasquier scale on axial T1 or FLAIR images. In cases where these sequences were not available, T2 images were used. For both MTA and GCA, there was good agreement between all sequences used (MTA κ=0.77; GCA κ=1.00). For both MTA and GCA, the hemisphere contralateral to the ICH was preferentially rated.

ICH location was defined as infratentorial, deep, or lobar, with the latter in cortical or cortical–subcortical regions and not involving any of the deep grey matter structures. Hematoma volume was calculated (S.L.) using a previously described validated semiautomated planimetric method.^21^

A clinico-radiological diagnosis of probable CAA was based on meeting the modified Boston criteria.^6^

The CAA score was calculated from a previously described 6-point scale.^7^ This scale awards 1 point for CSO-PVS rating of frequent-to-severe grades (ie, presence of >20 CSO-PVS) and WMH that is either Fazekas grade 3 if periventricular, or Fazekas grade ≥2 if deep.^22^ Additional points are awarded for the presence of lobar microbleeds (1 point if 2–4 are present; 2 points if there are ≥5) and cSS (1 point if focal; 2 points if disseminated).^7^

The SVD score was determined using a previously described 4-point scale.^22,23^ This scale awards 1 point for the presence of lacunes, microbleeds, BG-PVS rating of moderate-to-severe grades (ie, presence of >10 BG-PVS), and WMH that is either Fazekas grade 3 if periventricular or Fazekas grade ≥2 if deep.^22^

### Statistics

We investigated for selection bias within our final cohort by comparing the characteristics of people with appropriate MRI and those without. IQCODE was dichotomized using a cutoff of 3.3, and baseline characteristics were compared (Table 1) for those with scores >3.3 (ie, with cognitive impairment) and those with scores ≤3.3. Continuous data were reviewed for normality, and if normally distributed we used the independent *t* test. Where continuous variables were not normally distributed, we used the (nonparametric) Mann–Whitney *U* test. We used the χ^2^ tests for categorical variables. The independent *t* test (normally distributed continuous data) and the 2-sample test of proportion (categorical data) were used to compare means and proportions, respectively.

**Table 1.**
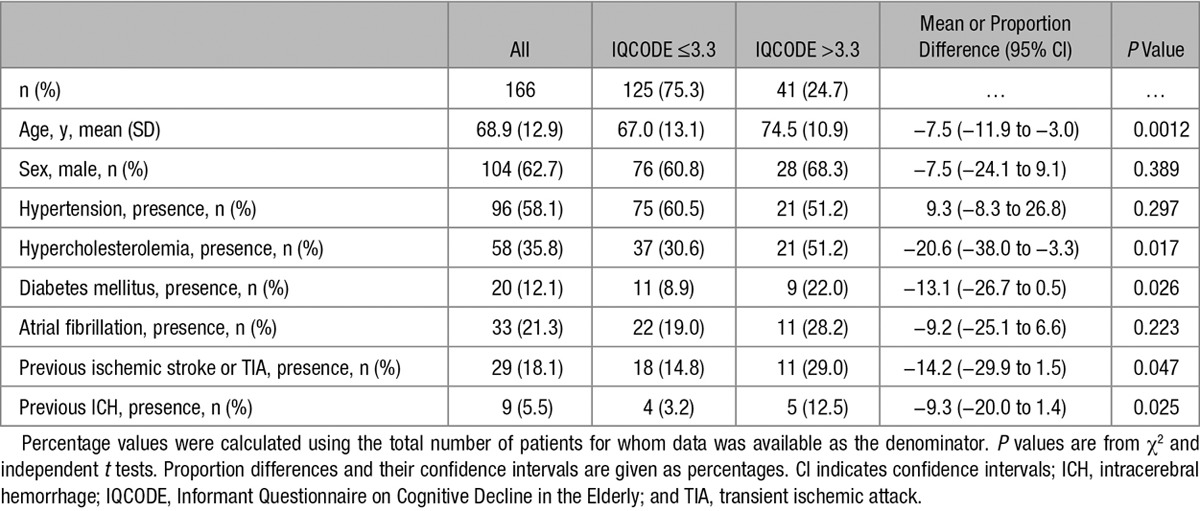
Baseline Demographic and Clinical Characteristics

Univariate comparisons were used to identify potential confounders for inclusion in the multivariable models; all variables with *P*<0.05 were included. We then performed adjusted logistic regression analyses, adjusting for significant associations identified in univariate analyses (Table 2). In further analyses (Table 3), we investigated associations with other neuroimaging markers suggestive of CAA (the presence of strictly lobar microbleeds, and presentation with lobar ICH), as well as a composite SVD score and its component elements. In these analyses, each neuroimaging marker was considered individually (ie, each adjusted model included only 1 neuroimaging marker at a time). Given that these analyses were exploratory, we did not make an adjustment for multiple testing.

**Table 2.**
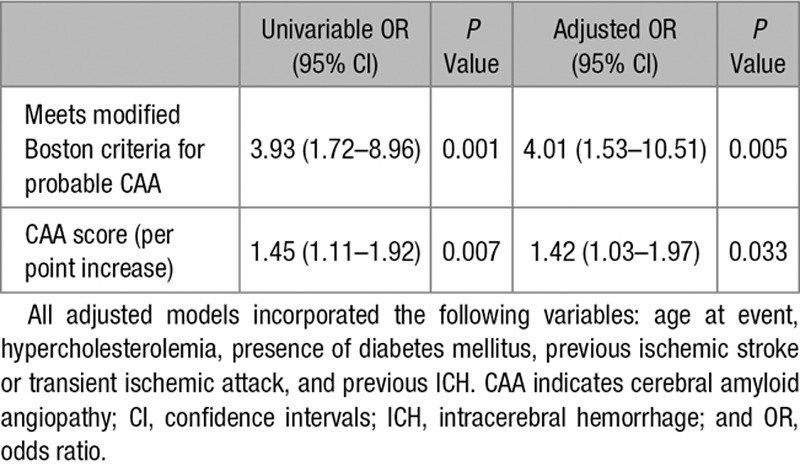
Univariable and Adjusted Logistic Regression Models, Investigating Associations Between Cognitive Impairment Before ICH and Evidence of CAA

**Table 3.**
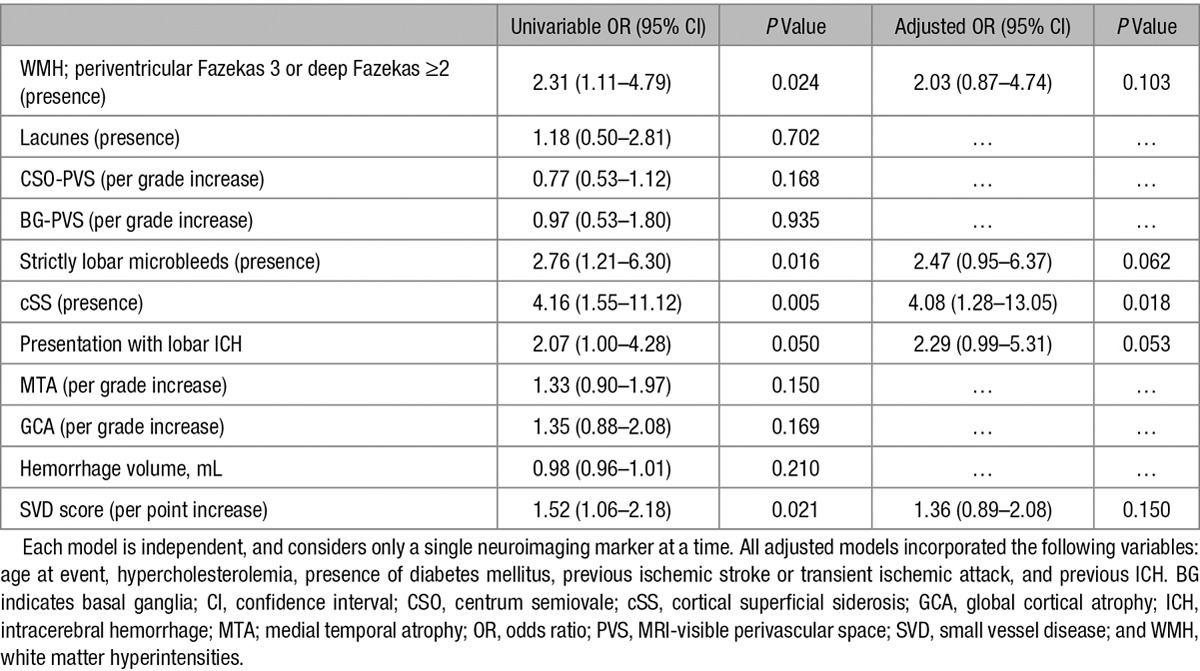
Logistic Regression Models (Univariable and Adjusted), Reviewing Associations Between Cognitive Impairment Before ICH and Individual Structural Markers of Cerebral SVD, and a Composite SVD Score

Statistical analysis was performed (G.B.) using Stata (Version 11.2).

## Results

### Cohort Characteristics

The demographic and imaging characteristics of those included (n=166) are shown in Table 1. Patients without MRI (n=588) and those with MRI but with missing or uninterpretable sequences (n=43) were excluded (online-only Data Supplement). When compared with the excluded patients (online-only Data Supplement), those included were younger (mean, 68.9 versus 75.0 years; *P*<0.00001), less likely to have hypertension (58.2% versus 70.9%; *P*=0.002), hypercholesterolemia (35.8% versus 47.9%; *P*=0.006), diabetes mellitus (12.1% versus 19.8%; *P*=0.024), and atrial fibrillation (12.3% versus 43.5%; *P*<0.0001), and more likely to have previously had an ischemic stroke or transient ischemic attack (24.7% versus 18.1%; *P*=0.081), lower Glasgow Coma Scale at presentation (interquartile range, 13–15 versus 14–15; *P*=0.003) and pre-ICH cognitive decline (38.2% versus 24.7%; *P*=0.001).

When comparing those with and without pre-ICH cognitive decline, those with (n=41) were older (mean difference, 7.5 years; *P*<0.0012) and more likely to have hypercholesterolemia (51.2% versus 30.6%; *P*=0.017), diabetes mellitus (22.0% versus 8.9%; *P*=0.026), previous ischemic stroke or transient ischemic attack (29.0% versus 14.8%; *P*=0.047), and previous ICH (12.5% versus 3.2%; *P*=0.025).

### Associations With Pre-ICH Cognitive Decline: Univariate and Multivariate Analyses

Univariate logistic regression analyses showed that pre-ICH cognitive decline was associated with meeting the modified Boston criteria for probable CAA at presentation and increasing CAA score (Table 2). In our multivariable analysis, we adjusted for age at event, hypercholesterolemia, presence of diabetes mellitus, previous ischemic stroke or transient ischemic attack, and previous ICH, which were statistically significant in univariate analyses (Table 1). Meeting the modified Boston criteria for probable CAA at presentation (odds ratio [OR], 4.01; 95% confidence interval [CI], 1.53–10.51); *P*=0.005) and increasing CAA score (for each point increase, OR, 1.42; 95% CI, 1.03–1.97; *P*=0.033) remained associated with pre-ICH cognitive decline (Table 2).

We then performed further analyses investigating the associations between individual neuroimaging markers of SVD and cognitive impairment before ICH. In univariable analyses (Table 3), we identified associations between pre-ICH cognitive decline and increasing SVD score, WMH, the presence of cSS, presence of strictly lobar microbleeds, and lobar ICH at presentation. In analyses adjusted for clinical and demographic variables identified in the univariate analysis (as above), the presence of cSS (OR, 4.08; 95% CI, 1.28–13.05; *P*=0.018), strictly lobar microbleeds (OR, 2.47; 95% CI, 0.95–6.37; *P*=0.062), and lobar ICH at presentation (OR, 2.29; 95% CI, 0.99–5.31; *P*=0.053) showed associations with pre-ICH cognitive impairment. The previous associations with increasing SVD score and WMH were no longer statistically significant, although for WMH a large effect size remained (OR, 2.03).

## Discussion

Our main new finding is that MRI neuroimaging markers of CAA are associated with pre-ICH cognitive impairment. This suggests that cognitive impairment in CAA is not only because of brain injury caused directly by ICH but also independently related to the underlying small vessel disruption associated with CAA.

Our findings add to growing evidence that CAA plays an important role in the development of cognitive impairment and dementia in those with ICH. The prevalence of pre-ICH dementia in lobar ICH is near double that in deep ICH,^24^ and structural imaging markers of CAA (cSS, cerebral microbleeds) present at the time of ICH are associated with later progression to dementia.^2^ Our results show that a composite CAA score has a per point association with cognitive decline; further studies could help establish whether such a score might be useful in patients with milder CAA (including those not fulfilling Boston criteria, or without macrohemorrhage). We found a strong association between cSS and pre-ICH cognitive impairment, suggesting that leptomeningeal hemorrhage, rather than parenchymal microbleeds, might be an especially important pathological process impairing cognition in CAA. Our findings also contribute to our understanding of the mechanisms by which CAA disrupts cognition, which include hematoma damage (via direct effects on cortical integrity and function^2^) and small vessel mechanisms. The latter may include effects on brain network efficiency,^25^ which correlates with cognitive performance and shows disturbances in the non-ICH hemisphere.^26^ Our finding that CAA is associated with cognitive impairment before ICH shows that hematoma damage cannot be the only mechanism contributing to cognitive disruption and supports the hypothesis that small vessel mechanisms are important.

A further possibility is that cognitive impairment before ICH is because of coincident Alzheimer’s disease.^4^ Although the co-occurrence of CAA and Alzheimer’s disease pathology is well recognized,^27^ CAA seems to have a cognitive profile distinct from that seen in Alzheimer’s disease, characterized primarily by deficits in processing speed and executive function.^28,29^ Recent neuropathological work^30^ found that CAA makes an independent contribution to cognitive performance in Alzheimer’s disease. Together, this evidence suggests that CAA has a specific neurovascular impact on cognitive performance, independent of coexistent Alzheimer’s pathology. Although we did not find an association between MTA or GCA (as putative imaging markers of Alzheimer’s pathology^31^) and pre-ICH cognitive impairment, we acknowledge that our sample size is small and so we cannot rule out missing subtle effects.

The main strength of this study is our detailed neuroimaging description of the structural markers of cerebral SVD in the context of pre-ICH cognitive decline, in a richly phenotyped prospective nationwide cohort of patients. However, our work also has limitations. Those included in our study were younger, with fewer comorbidities and a lower IQCODE than those who did not have an interpretable MRI; additionally, we acknowledge that a suspicion of CAA could increase the likelihood of an MRI being performed (50% of our included patients presented with lobar ICH), and so our final cohort might not be representative of those presenting with a spontaneous ICH to an acute stroke service. Brain imaging at each study center was completed according to local protocols, and so there are unavoidable variations in the nature and manner of the sequences obtained, which could influence our results. In particular, the use of susceptibility-weighted versus T2*-weighted gradient echo sequences may result different microbleed counts, as the former is more sensitive to this; we did not adjust for this in our analyses. There are inherent limitations of using the IQCODE, including variations in the threshold used to define cognitive impairment and the lack of validation against a reference standard for prestroke cognitive impairment. Finally, we acknowledge that our study size is small, and so our results should be interpreted cautiously, particularly the adjusted analyses. As detailed, we chose not to apply an adjustment for multiple testing in order not to miss potential associations of interest. In addition, although our study is powered to detect moderate effect sizes, it may have missed smaller effects.

Cognitive impairment before ICH is common and is associated with imaging findings consistent with an important contribution from CAA. This suggests that any future strategy aiming to reduce the impact of poststroke dementia in ICH will need to extend beyond stroke prevention and include strategies that address the small vessel impact of CAA. Further work on the natural history of when and how CAA may influence an individual’s cognitive profile is a priority for future research.

## Appendix

The CROMIS-2 Collaborators: Louise Shaw, MD; Jane Sword, MD; Azlisham Mohd Nor, MD; Pankaj Sharma, PhD; Roland Veltkamp MD; Deborah Kelly, MD; Frances Harrington, MD; Marc Randall, MD; Matthew Smith, MD; Karim Mahawish, MD; Abduelbaset Elmarim, MD; Bernard Esisi, MD; Claire Cullen, MD; Arumug Nallasivam, MD; Christopher Price, MD; Adrian Barry, MD; Christine Roffe, MD; John Coyle, MD; Ahamad Hassan, MD; Caroline Lovelock, DPhil; Jonathan Birns, MD; David Cohen, MD; L. Sekaran, MD; Adrian Parry-Jones, PhD; Anthea Parry, MD; David Hargroves, MD; Harald Proschel, MD; Prabel Datta, MD; Khaled Darawil, MD; Aravindakshan Manoj, MD; Mathew Burn, MD; Chris Patterson, MD; Elio Giallombardo, MD; Nigel Smyth, MD; Syed Mansoor, MD; Ijaz Anwar, MD; Rachel Marsh, MD; Sissi Ispoglou, MD; Dinesh Chadha, MD; Mathuri Prabhakaran, MD; Sanjeevikumar Meenakishundaram, MD; Janice O’Connell, MD; Jon Scott, MD; Vinodh Krishnamurthy, MD; Prasanna Aghoram, MD; Michael McCormick, MD; Paul O’Mahony, MD; Martin Cooper, MD; Lillian Choy, MD; Peter Wilkinson, MD; Simon Leach, MD; Sarah Caine, MD; Ilse Burger, MD; Gunaratam Gunathilagan, MD; Paul Guyler, MD; Hedley Emsley, MD; Michelle Davis, MD; Dulka Manawadu, MD; Kath Pasco, MD; Maam Mamun, MD; Robert Luder, MD; Mahmud Sajid, MD; Ijaz Anwar, MD; James Okwera, MD; Julie Staals, PhD; Elizabeth Warburton, MD; Kari Saastamoinen, MD; Timothy England, MD; Janet Putterill, MD; Enrico Flossman, MD; Michael Power, MD; Krishna Dani, MD; David Mangion, MD; Appu Suman, MD; John Corrigan, MD; Enas Lawrence, MD; and Djamil Vahidassr, MD.

## Sources of Funding

The CROMIS-2 study is funded by the Stroke Association and British Heart Foundation. G. Banerjee receives funding from the Rosetrees Trust. Dr Ambler receives funding from the National Institute for Health Research University College London Hospitals Biomedical Research Centre. Dr Al-Shahi Salman is funded by an Medical Research Council senior clinical fellowship. M.M. Brown’s Chair in Stroke Medicine is supported by the Reta Lila Weston Trust for Medical Research. Dr Werring receives research support from the Stroke Association, the British Heart Foundation, and the Rosetrees Trust. This work was undertaken at University College London Hospitals and University College London which receive a proportion of funding from the Department of Health National Institute for Health Research (NIHR) Biomedical Research Centres funding scheme.

## Disclosures

Dr Cohen has received institutional research support from Bayer; honoraria for lectures and an Advisory Board from Bayer, diverted to a local charity; and travel/accommodation expenses for participation in scientific meetings covered by Bayer and Boehringer Ingelheim. G.H.Y. Lip has served as a consultant for Bayer, Astellas, Merck, AstraZeneca, Sanofi, BMS/Pfizer, Biotronik, Portola, and Boehringer Ingelheim and has been on the speakers’ bureau for Bayer, BMS/Pfizer, Boehringer Ingelheim, and Sanofi-Aventis. The other authors report no conflicts.

## Supplementary Material
